# Overparenting, Parent-Child Conflict and Anxiety among Chinese Adolescents: A Cross-Lagged Panel Study

**DOI:** 10.3390/ijerph182211887

**Published:** 2021-11-12

**Authors:** Janet Tsin-Yee Leung

**Affiliations:** Department of Applied Social Sciences, The Hong Kong Polytechnic University, Hung Hom, Kowloon, Hong Kong HJ402, China; janet.leung@polyu.edu.hk

**Keywords:** overparenting, parent-child conflict, anxiety, adolescents, cross-lagged panel study, Chinese

## Abstract

Background: Overparenting is an emerging parenting style in which parents over-protect their children from difficulties and challenges by intruding into their lives and providing extensive assistance to them. Unfortunately, longitudinal studies related to overparenting were severely lacking, particularly on its impacts on early adolescents. Moreover, studies examining the mediational pathways through which overparenting is associated with adolescent anxiety are scant. This study examined the mediating role of parent-child conflict (father-child and mother-child) in the relationship between overparenting (paternal and maternal) and adolescent anxiety over time. Method: Based on a three-wave longitudinal data of 1074 Chinese early adolescents in Hong Kong, the relationships among paternal and maternal overparenting, father- and mother-child conflict, and adolescent anxiety were assessed. Results: Mother-child conflict mediated the relationship between maternal overparenting and adolescent anxiety over time. Besides, a reverse association of prior adolescent anxiety with subsequent maternal overparenting via mother-child conflict was also identified. In addition, adolescent gender and family intactness did not moderate the relationships among overparenting, parent-child conflict, and adolescent anxiety. Discussion: This present study identified that the bidirectional relationship between maternal overparenting and adolescent anxiety via mother-child conflict over time, which sheds new light on the study of overparenting on adolescent well-being in the Chinese communities.

## 1. Introduction

In the past decade, overparenting has caught the attention of researchers, educators, and family theorists in understanding the relationship between overparenting and development and wellbeing among emerging adults [1,2,3,4]. Overparenting is a developmentally inappropriate parenting style where parents overprotect their children by involving too much in their daily lives and providing tremendous care and attention to them [4]. Though many parents who practice overparenting start from goodwill to ensure their children’s success, competence, and happiness [5,6], empirical evidence consistently shows that overparenting is associated with adolescents’ negative outcomes, including increasing adolescent anxiety [6,7], emotional distress [8,9], procrastinated behavior [10] and narcissistic traits [4,11], and decreasing adolescents’ academic motivation [12], life satisfaction [3] and self-efficacy [13,14]. However, the study of overparenting is still at the infancy stage. There are several limitations in the existing literature. First, a vast majority of studies adopted a cross-sectional approach in research design e.g., [4,12], which fails to examine the associations of overparenting with adolescent development and wellbeing over time. Assessing the reverse effects of adolescent development on overparenting is indeed impossible. Along the same vein, some studies examined the mechanisms through which the relationship between overparenting and adolescent development occurred. For example, Rousseau and Scharf [2] found that attachment anxiety and psychological control partially mediated the linkage between paternal overparenting and poor adjustment of emerging adults. Segrin et al. [6] also found that overparenting was associated with more ineffective coping skills of young adult children, which in turn was associated with greater stress and anxiety. However, these scholars admitted the limitations of cross-sectional studies in supporting or refuting mediational effects without a temporal ordering of predictor, mediator, and outcome variables [2,6]. Indeed, examination of the underlying mechanisms in the relationship between overparenting and adolescent developmental outcomes is noteworthy. Second, the study of overparenting and helicopter parenting has focused mainly on emerging adults, while overparenting on young adolescents has been largely ignored [11]. Overparenting practice will never be developed in a nutshell. Parents who practice overparenting are anxious about their children’s development when their children are young [15]. Overparenting may be salient for parents with early adolescents when they need to modify their parenting practice and readjust their parent-child relationship boundaries [16]. However, some parents may retain the parenting strategies that they have used in their children’s childhood to rear their adolescent children. Moreover, some parents are anxious and less tolerant when they anticipate higher risks and vulnerabilities for their children during the individuation process in adolescence [17], and hence they over-react to protect their children from potential developmental vulnerabilities and failures [11]. Unfortunately, studies on overparenting for early adolescents are very limited. Third, culture plays an important role in shaping parenting practice [18]. Hence, cultural specificity needs to be emphasized in the conceptualization of overparenting practice. In Chinese culture where family pride is regarded as a central pillar of Chinese familism [19], Chinese parents are obligated to ensure their children’s success and achievement to bring honor to the family [20]. Though Hong Kong has been influenced by western culture as it was formerly a British colony, family pride is still the core belief of Chinese families in Hong Kong [21]. In a qualitative study exploring how Chinese parents and adolescents perceived overparenting, both parent and adolescent informants suggested that parental over-reaction on children’s academic performance, over-scheduling of tutorials and talent enhancement classes, and frequent comparison of children’s achievement among peers were features of overparenting specific to Chinese [22]. Thus, it is necessary to use culturally sensitive measurements in the study of overparenting in Chinese communities. Last but not the least, few studies examined the relative contributions of paternal and maternal overparenting in their associations with adolescent wellbeing and development e.g., [4,23]. In fact, fathers are more likely to take up the bread-winning role in the family, whereas mothers are responsible for child-rearing and family management in Chinese families [24]. Gender role differences in family processes and parenting may contribute to different impacts on adolescent development [25]. Hence, it is commendable to examine paternal and maternal overparenting as two separate variables. As such, this study examined the relationships among paternal and maternal overparenting, father-child and mother-child conflict, and adolescent anxiety using three-wave longitudinal data of Chinese adolescents in Hong Kong.

### 1.1. Overparenting in Chinese Contexts

Overparenting is defined as “a form of developmentally inappropriate parenting that is driven by parents’ overzealous desires to ensure the success and happiness of their children, typically in a way that is construed largely in the parents’ terms, and to remove any perceived obstacles to those positive outcomes” (4, p. 238). Segrin et al. [4] further identified four distinct characteristics of overparenting, namely anticipatory problem-solving and risk aversion, excessive advice and affective response, dominance over children’s self-direction, and excessive tangible assistance to their children. Regarding the different conceptualizations of overparenting in both Chinese and U.S. cultures, Leung et al. [22] conducted a qualitative study to explore the features of overparenting from the perspectives of parents and adolescents respectively. Eight features were extracted from focus group interviews, namely close monitoring and surveillance, intrusion of children’s daily routine and life direction, an over-emphasis of children’s school achievements, repeated comparisons of children’s performance with peers, overscheduling of children’s activities, anticipatory problem-solving, excessive affective involvement and excessive care [22]. While some characteristics are similar to those identified by Segrin et al. [4] (e.g., anticipatory problem solving, excessive advice and care, dominance over children’s self-direction), some are unique characteristics addressing the supreme goals of family pride embedded in Chinese culture [26] (e.g., a strong emphasis on children’s school achievements, repeated comparisons of children’s performance with peers, and overscheduling of children’s activities). Corresponding to Baumrind’s [27] framework of parenting styles, Leung & Shek [28] found that the eight overparenting features were subsumed under a hierarchical two-factor structure: over-demandingness and over-responsiveness. Overparenting is not only employed in socializing emerging adults, similar features and patterns of overparenting are identified in parenting young adolescents [28].

### 1.2. Overparenting, Parent-Child Conflict, and Adolescent Anxiety

Attachment theory [28] explains the relationship between overparenting and anxiety among Chinese adolescents. According to attachment theory, a secure platform is necessary for children to explore the outside world [29]. However, parents who practice overparenting may hinder their children from progressive independent development, which may lead to anxious attachment characterized by immaturity, anxiety, and depression [30]. Indeed, evidence showed that higher levels of overparenting are associated with poorer psychological wellbeing [8] and life satisfaction [3] among adolescents, and with greater adolescent depression and anxiety [2,3,7].

Self-determination theory [31] further accounts for the mediating role of parent-child conflict in the relationship between overparenting and adolescent anxiety. According to the theory, three basic psychological needs, namely autonomy, relatedness, and competence, are linked with each other to guide human behavior [31]. During adolescence, young people seek more autonomy and independence in their search for self-identity [32]. Overparenting involves parental intrusion and overprotection, which may hinder adolescents’ desire for autonomy and differentiation from their parents [33]. Adolescents may resist parental intrusion by acting against their parents’ wishes, which will generate parent-child conflict. A poorer parent-child relationship may in turn lead to poorer adolescent wellbeing [34].

According to the transactional theory of human development [35], the linkage between parental behavior and adolescent development is bi-directional in nature, i.e., parental behavior has impacts on adolescent developmental attributes and wellbeing, at the same time, adolescent attributes and wellbeing also affect parents in their choice of parenting strategies. Evidence supports the bi-directional relationship between parental behavior and adolescent development e.g., [36,37,38]. For instance, based on a longitudinal study on parental sacrifice and hopelessness among Chinese adolescents in Hong Kong, bi-directional effects were identified in the relationship between maternal sacrifice and adolescent hopelessness, through which filial piety served as a mediator [36]. When adolescents experience stress and anxiety, they may easily spill over their emotions to their parents, which may heighten parent-child conflict. Parents may overreact to their children’s behavior and emotions by exercising over-control and over-protection. Evidence shows that parents with anxious children demonstrated more parental negativity and involvement to their children [39,40]. Unfortunately, the adoption of cross-sectional studies fails to examine the bi-directional linkage between overparenting and adolescent development in the existing literature. Thus, it is essential to examine bi-directional relationships among overparenting, parent-child conflict, and adolescent anxiety using longitudinal data.

### 1.3. Parent Gender, Adolescent Gender and Family Intactness in Contributing to the Relationships

Parent gender may contribute to the relationship among overparenting, parent-child conflict, and adolescent anxiety. According to the sex role theory [41], fathers are more goal-oriented and achievement-focused in parenting their children, whereas mothers are more affective and relational in socializing their children [42]. Hence, maternal parenting practice is more influential to the changes in the parent-child relationship and their children’s emotional wellbeing [43].

For adolescent gender, girls are found to be more sensitive to parental affection than are boys [44,45]. Evidence showed that overparenting was detrimental to girls’ wellbeing than to boys’ [46], and maternal overprotection was positively linked to girls’ somatic symptoms than to boys’ [47]. However, there were studies showing that adolescent gender did not moderate the relationship between overparenting and adolescents’ wellbeing [48,49]. Hence, it is insightful to examine whether the relationships among overparenting, parent-child conflict, and adolescent wellbeing will be different between adolescent boys and girls.

Family intactness may contribute to the relationships among overparenting, parent-child conflict, and adolescent anxiety. In the studies on the relationship between marital quality and parenting strategies, the spillover effect and the compensatory effect are controversial in family research e.g., [50,51]. The spillover hypothesis posits that parents with marital discord may transfer their negative emotions created in the marital subsystem to the parent-child subsystem, leading to a poorer parent-child relationship and higher levels of harsh parenting [50,52]. On the contrary, the compensatory hypothesis suggests that parents who have experienced marital discord may compensate their children for the loss or deficiencies [40,53], and parents become more supportive of their children’s wellbeing and development [54]. Evidence shows that fathers are more vulnerable to spilling over their stresses from marital discord to more negative parenting [51], whereas mothers tend to compensate for their children by giving more support [54]. When applying to overparenting and adolescent wellbeing, a previous study showed support that maternal overparenting reduced adolescent depression in single-parent families than those in intact families, supporting the compensatory hypothesis [7].

### 1.4. The Present Study

The present study examined the bi-directional mediating effects of father-child conflict and mother-child conflict in the relationships between paternal and maternal overparenting and adolescent anxiety over time in a sample of Chinese adolescents in Hong Kong.

**Hypothesis** **1a** **(H1a).** *It was hypothesized that maternal overparenting would be associated with more mother-child conflict, which in turn would increase adolescent anxiety over time.*

**Hypothesis** **1b** **(H1b).** *There would be a reverse indirect effect of mother-child conflict on the relationship between prior adolescent anxiety and subsequent maternal overparenting over time.*

The moderating effects of adolescent gender and family intactness in the relationship among overparenting, parent-child conflict, and adolescent anxiety over time were also examined. It was hypothesized that adolescent gender and family intactness would moderate the relationship between maternal overparenting and adolescent anxiety over time.

**Hypothesis** **2** **(H2).** *Girls would perceive higher levels of anxiety when there would be higher levels of maternal overparenting than would boys.*

**Hypothesis** **3** **(H3).** *At higher levels of maternal overparenting, it was hypothesized that adolescents would perceive lower levels of anxiety in non-intact (divorced, widowed, separated, and remarried) families than would those in intact families.*

## 2. Materials and Methods

### 2.1. Participants and Procedures

The respondents were recruited from secondary schools using a multi-stage stratified cluster sampling method, with geographical location and school banding as the stratifying factors. 14 secondary schools across Hong Kong joined the study and completed the questionnaires at three time points. At Time 1 (T1), 1463 students were studying Form One (Grade 7) participated in the study. The students were requested to fill out the questionnaire at Time 2 (T2) and Time 3 (T3), with an interval of one year. However, due to the outbreak of the COVID-19 pandemic that classes were suspended in the first six months of 2020, data was collected during Semester One of Form 4 (Grade 10) at T3. After matching, 1074 respondents were filling out the questionnaires at all three time points. The attrition rate was calculated by dividing the number of respondents who joined the study at T1 but failed to complete the survey at T2 and/or T3 by the total number of respondents at T1. The attrition rate of this study was 26.6%.

At T1, 685 (46.8%) respondents were girls. The mean age was 12.66 (SD = 0.80). A majority of respondents came from intact families (n = 1082; 74.0%), while 315 (21.6%) respondents coming from non-intact families, including remarried (n = 119; 8.1%), divorced (n = 108; 7.4%), separated (n = 55; 3.8%) and widowed (n = 33; 2.3%) families [n = 20 respondents reported others (living with relatives, did not know), and n = 46 did not respond]. 292 (20.0%) respondents were recipients of Comprehensive Social Security Assistance (CSSA), which is a means-tested cash assistance for those families living in poverty. It should be noted that the percentage of CSSA recipient respondents in this sample was greater than that of the official statistics (In 2020, 8.5% of total households were CSSA recipients in Hong Kong) [55,56].Logistic regression analyses were performed to assess whether demographic characteristics (i.e., adolescent gender, family intactness) and the studied variables (i.e., perceived paternal and maternal overparenting, father-child and mother-child conflict, and anxiety) at Time 1 were associated with sample attrition (dropout = 0, retention = 1). Results showed significant associations of sample retention with adolescent gender (odds ratio [OR] = 1.27, *p* < 0.001) and family intactness (odds ratio [OR] = 1.20, *p* < 0.05), with more girls and adolescents from intact families retained in the study. However, the associations of all studied variables at T1 with sample retention were non-significant, suggesting that there was a random dropout of the respondents [57]. In this study, a sample of 1,074 respondents who had filled out the questionnaires at all three time points was used in the analyses.

Before data collection, invitation letters on objectives and procedures of the study were sent to students’ parents via secondary schools. Written informed consent was obtained from the parents. The data collection was conducted during class lessons. The research objectives, anonymity principles in data collection procedures, and respondents’ rights to voluntarily participate in and withdraw from the research were explained by trained research assistants. Written consent from the respondents was obtained. The students filled out a questionnaire that contained measures of perceived parental and maternal overparenting, father-child and mother-child conflict, anxiety, and some demographic questions (e.g., gender, family intactness). Those students who did not join the research were allowed to do their home assignments in class. The respondents were given adequate time to complete the questionnaires. Identical procedures were repeated in T2 and T3. All participants and their parents gave their informed consent for inclusion before they participated in the study. The study was conducted in accordance with the Declaration of Helsinki, and the protocol was approved and monitored by the Human Subjects Ethics Sub-committee of The Hong Kong Polytechnic University (Project identification code: HSEARS20170126002).

### 2.2. Instruments

#### 2.2.1. Overparenting

Chinese Paternal and Maternal Overparenting Scales (PCOS/MCOS). Overparenting is a composite of various forms of over-demanding and over-responding parenting practice [28]. Based on the existing overparenting literature e.g., [4] as well as the qualitative results of parents’ and adolescents’ focus groups on Chinese overparenting [11], the 42-item PCOS/MCOS were developed with eight dimensions: parental surveillance, parental intrusion into children’s routines and direction, over-emphasis on children’s school performance, repeated comparisons of children’s achievements with other children’s, over-scheduling of tutorials and talent enhancement classes, anticipatory problem-solving, excessive care, and disproportionate affective involvement [58]. Over-emphasis on children’s school performance and over-scheduling of tutorials and talent enhancement classes are regarded as unique features of concerted cultivation specific to Chinese parenting practice [34]. A sample item reads “I need to report everything to my father/mother” Respondents rated each item on a 6-point Likert scale (1 = “strongly disagree”; 6 = “strongly agree”). Both PCOS and MCOS showed good psychometric properties in a sample of early Chinese adolescents [24]. Higher mean scores in PCOS and MCOS represent stronger perceived paternal and maternal overparenting, respectively. Both PCOS and MCOS showed excellent reliability in this study (PCOS: α at Time 1 = 0.95; α at Time 2 = 0.95; α at Time 3 = 0.97; MCOS: α at Time 1 = 0.95; α at Time 2 = 0.96; α at Time 3 = 0.97).

#### 2.2.2. Parent-Child Conflict

Father–Adolescent Conflict and Mother–Adolescent Conflict Scales (FAC/MAC). Shek [59] modified the Conflict Behavior Questionnaire [60] to develop the Chinese FAC/MAC. Both FAC and MAC showed good psychometric properties in previous studies [59,61]. A three-item short form was used in this study [61]. A sample item reads “My father and I always criticize or pick on each other.” Respondents rated each item on a 6-point Likert scale (1 = “strongly disagree”; 6 = “strongly agree”). Higher mean scores in FAC and MAC indicate greater father-child and mother-child conflict, respectively. Both FAC and MAC showed good reliability in this study (FAC: α at Time 1 = 0.89; α at Time 2 = 0.90; α at Time 3 = 0.91; MAC: α at Time 1 = 0.90; α at Time 2 = 0.89; α at Time 3 = 0.90).

#### 2.2.3. Adolescent Anxiety

Anxiety Subscale (AS_HADS-C) of Chinese Hospital Anxiety and Depression Scale (HADS-C). Based on the Hospital Anxiety and Depression Scale HADS; [62], Leung and colleagues [63] translated the measurement into the Chinese version (HADS-C). The Anxiety Subscale (7 items) was used in the study. A sample item reads “I get sudden feelings of panic”. Respondents rated each item on a 4-point Likert scale (0 = “not at all” to 3 = “most of the time”). Higher mean scores of the AS_HADS-C indicate higher levels of anxiety. The AS_HADS-C showed acceptable reliability of the study (α at Time 1 = 0.74; α at Time 2 = 0.76; α at Time 3 = 0.76).

### 2.3. Data Analyses

To test the bi-directional mediating roles of father-child conflict and mother-child conflict in the relationship between perceived paternal and maternal overparenting and adolescent anxiety over time, cross-lagged panel analyses using the software program of AMOS 26.0 were performed [64]. NFI and CFI > 0.90 and RMSEA < 0.08 were used as the goodness-of-fit indicators [65]. I further performed bootstrapping mediation tests using 5000 bootstrapped re-samples in AMOS 26.0 to assess the significance of the mediating effects [66]. In case a “zero” value did not appear between the upper and lower bounds of bias-corrected 95% confidence intervals in the bootstrapping mediation test, the mediating effect was supported [66]. The ratio of indirect effect to the total effects was regarded as the effect size of the standardized indirect effect [67]. In case direct and indirect effects show opposite direction, absolute values of regression coefficients were used in calculating the ratio [68].

In addition, multiple group analyses were performed to assess whether adolescent gender and family intactness would moderate the regression paths among paternal and maternal overparenting, father-child and mother-child conflict, and adolescent anxiety. The unconstrained model was evaluated using NFI and CFI > 0.90, RMSEA < 0.08 as the goodness-of-fit indicators [65]. An equality constraint was imposed on each structural path between groups. In case the chi-square value of an imposed constraint was significant between the tested model and the unconstrained model, a moderating effect of the moderator was supported.

## 3. Results

Table 1 lists the descriptive statistics of the studied variables. The absolute values of skewness and kurtosis were less than 2 and 7 respectively, supporting the assumptions of normality of the studied variables [69]. Full information maximum likelihood (FIML) estimation was adopted in handling missing data. FLML is a technique to estimate each parameter directly without filling in missing data values. It estimates the likelihood function for each respondent based on one’s available variables [70]. This technique is robust in accommodating missing data and enhancing the accuracy and power of analyses when compared with other approaches [71]. Correlational analyses showed that perceived paternal overparenting at T1 was positively related to father-child conflict concurrently and longitudinally, but it was only related to anxiety at T1. Perceived maternal overparenting at T1 was positively related to mother-child conflict and anxiety concurrently and longitudinally. Furthermore, adolescent anxiety at T1 was positively related to maternal overparenting, father-child conflict, and mother-child conflict concurrently and longitudinally, and it was associated with paternal overparenting at T1. In addition, boys perceived more negatively on paternal overparenting and father-child conflict than did girls at all three time points (Table 2). Family intactness was also positively related to paternal overparenting at three time points but was negatively related to mother-child conflict at T1 and T3 respectively (Table 2).

**Figure 1:**
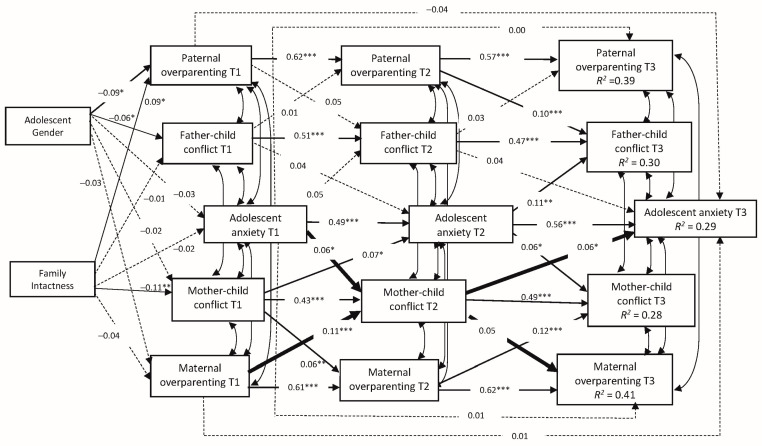
Cross-lagged model of the relationship among paternal and maternal overparenting, father-child conflict and mother-child conflict, and adolescent anxiety. * *p* < 0.05, ** *p* < 0.01, *** *p* < 0.001.

We tested the bi-directional mediational model (M1), which included the direct effect of paternal and maternal overparenting at T1 on adolescent anxiety at T3, the reverse direct effect of adolescent anxiety at T1 on paternal and maternal overparenting at T3, and bi-directional indirect effects with father-child conflict and mother-child conflict at T2 serving as mediators (Figure 1). M1 showed an acceptable fit of the data, with CFI and NFI values of 0.945 and 0.936 respectively [65]; and RMSEA value of 0.073 [65] (Table 3). Maternal overparenting at T1 was positively related to greater mother-child conflict at T2 (β = 0.11; *p* < 0.001), which in turn was associated with higher levels of adolescent anxiety at T3 (β = 0.06; *p* < 0.01). H1a was supported. Moreover, adolescent anxiety at T1 was associated with greater mother-child conflict at T2 (β = 0.06; *p* < 0.05), which in turn was linked to stronger maternal overparenting at T3 (β = 0.05; *p* < 0.05). H1b was also supported (Figure 1). Bootstrapping mediation tests [62] supported both mediating effects of mother-child conflict at T2 on the relationship between maternal overparenting at T1 and adolescent anxiety at T3 [standardized indirect effect = 0.007, *p* < 0.05; 95% CI = (0.001; 0.010); effect size = 0.40] and that on the relationship between adolescent anxiety at T1 and maternal overparenting at T3 [standardized indirect effect = 0.003, *p* < 0.05; 95% CI = (0.001; 0.016); effect size = 0.38].

For the moderating effects of adolescent gender on the bi-directional indirect relationships between overparenting (paternal and maternal) and adolescent anxiety via father-child and mother-child conflict, multiple group analyses did not show any difference between constrained (M2b) and unconstrained (M2a) models across both gender groups, with Δx^2^ = 41.564 (*p* > 0.05) (Table 3). H2 was not supported. Moreover, there was invariance in the tested model between adolescents from intact families and those from non-intact families (divorced, separated, widowed, and remarried families), with Δx^2^ = 40.324 (*p* > 0.05) between constrained (M3b) and unconstrained (M3a) models (Table 3). H3 was not supported. In summary, adolescent gender and family intactness did not moderate the indirect relationships between paternal and maternal overparenting and adolescent anxiety via father- and mother-child conflict.

## 4. Discussion

The study examined the relationships among paternal and maternal overparenting, father-child and mother-child conflict, and adolescent anxiety in Chinese adolescents in Hong Kong. Results indicated that mother-child conflict mediated the relationship between maternal overparenting and adolescent anxiety over time, supporting H1a. Furthermore, the mother-child conflict also mediated the reverse effects of prior adolescent anxiety on subsequent maternal overparenting over time, supporting H1b. The findings lend some support to self-determination theory [31] by showing that maternal overparenting increases adolescent anxiety via heightened mother-child conflict (i.e., H1a). Adolescents seek more autonomy and independence during the individuation process [17]. However, parents who practice overparenting fail to grant autonomy to their adolescent children and intrude into their daily routine and life direction [72]. Furthermore, parental overprotection also obstructs adolescents from self-differentiation from their parents [73]. The desire for greater autonomy and differentiation may lead to conflict with their parents. Indeed, parent-child conflict may reduce adolescents’ relatedness with their parents, which may link to greater anxiety. As predicted, the mediational pathway occurred at the maternal side. Paternal overparenting was not associated with adolescent anxiety over time. Based on gender role theory [74], mothers take up more responsibilities in family management and child-rearing, whereas fathers are mainly the breadwinners of the family. The proposition also corresponds to the Chinese cultural inclination of “nan zhu wai, nu zhu nei” (man works outside the family, whereas woman works inside the family) [75]. Hence, fathers are less involved in parenting (and overparenting) and paternal overparenting is less influential to induce anxiety in their adolescent children.

Interestingly, the results indicated that prior anxiety was associated with subsequent maternal overparenting via the induction of mother-child conflict (i.e., H1b). Anxious adolescents are more likely to lose their temper when they feel stressed, which may heighten parent-child conflict. In response, parents may become more over-involved and over-controlling to handle their children’s emotions [39], hoping to reduce their children’s anxiety and distress [76]. As mothers are more affective and responsive to their children’s needs [43], they may practice more overparenting to regulate their children’s emotions.

Although some cross-sectional studies identified that the association of overparenting with adolescent wellbeing was stronger for boys than girls e.g., [46], the findings showed that adolescent gender did not moderate the relationships among overparenting, parent-child conflict, and adolescent anxiety over time (i.e., H2). During the individuation process, adolescents need to separate themselves from their parents and explore their self-identity through interactions with the outside world [17,77], regardless of adolescent gender. Hence, both adolescent boys and girls may have a conflict with their overparenting parents during the adolescence stage. The findings also indicated that family intactness did not moderate the relationships among overparenting, parent-child conflict, and adolescent anxiety over time (i.e., H3). As mentioned, when adolescents develop, they seek greater autonomy for their development. Even though single parents may want to compensate their children by overprotecting them from further hurt and distress, adolescents from single-parent families may still prefer to have more autonomy and space to build up their self-identity and competence. Hence, overparenting practice may be regarded as a constraint on adolescents’ pursuit of autonomy and independence, regardless of family structure.

There are several implications in the study. Theoretically, the study is pioneering in examining the relationships among overparenting, parent-child conflict, and adolescent anxiety over time using longitudinal data of a sample of Chinese adolescents. The mediating role of mother-child conflict on the association of prior maternal overparenting and subsequent adolescent anxiety was identified. Moreover, a reverse association of prior adolescent anxiety with subsequent maternal overparenting via mother-child conflict was also confirmed. In view of the paucity of conducting longitudinal research on overparenting, the study provides a more robust empirical support of the mediating role of parent-child conflict in the bi-directional relationships between overparenting and adolescent anxiety, which facilitates the development of family process model related to overparenting. Furthermore, the study identified that the mediation occurred on the maternal side of Chinese families, whereas the father-child conflict did not mediate the relationship between paternal overparenting and adolescent anxiety. The findings provide insight on understanding how parent gender makes a difference in the effects of adolescent anxiety, which is essential in the study of adolescence and the family. Last but not the least, the findings indicated that adolescent gender and family intactness did not moderate the relationship among overparenting, parent-child conflict, and adolescent anxiety over time, which add new evidence to the study of overparenting in early adolescence.

Practically, while many studies indicate that negative parenting (e.g., parental alienation, harsh parenting, psychological control) lead to parent-child conflict and adolescent anxiety [77,78,79,80,81], it is insightful to identify that over-involvement and over-care of mothers is associated with higher adolescent anxiety via heightened mother-child conflict. Hence, family practitioners and youth counselors may need to help those families practicing overparenting. Parental education is essential to reduce mothers’ practice of overparenting, and family counseling can be provided for mother-child dyads to resolve their conflict.

There are several limitations of the study. First, the study was designed to capture adolescents’ perspectives in examining the relationship between overparenting, parent-child conflict, and anxiety. This approach is justified as adolescents are “receivers” of parenting practice [82] and their subjective reactions to overparenting are critical in inducing their emotions. However, as pointed out by Day et al. [83], collecting data from solely one member may result in limitations for “extrapolating a sequence of events that may lead to a certain decision or interactional style” (p. 110). Hence, it is methodologically preferable to collect data from multiple informants (i.e., parents and adolescents). Secondly, data collection at T3 was largely affected by the COVID-19 pandemic and school suspension. Evidence shows that routine disruption due to the pandemic is associated with greater parent-child conflict, which leads to the poor psychological wellbeing of children and adolescents [84]. The situation may be more salient for mothers who practice overparenting, as mothers may feel disturbed by routine disruption and they intrude more into their children’s lives to reduce their anxiety [15], which may heighten mother-child conflict. Hence, it is suggested to replicate the study in the future. Third, as the percentage of CSSA recipient respondents in the sample was greater than that of the official statistics in 2020 (i.e., 8.5% of total households were CSSA recipients in Hong Kong) [56,57], the findings should be interpreted with caution. Fourth, the adolescent sample was recruited in Hong Kong. Since Hong Kong was formerly a British colony and families are affected by both Chinese and Western cultures, the relationship among overparenting, parent-child conflict, and adolescent anxiety may be different from those in other Chinese communities. There is a need to replicate the study in other Chinese communities (such as mainland Taiwan, China) and non-Chinese communities (e.g., the U.S.). Last but not the least, as overparenting has been identified in emerging adults, it is suggested to examine the relationships among overparenting, parent-child conflict, and adolescent anxiety in a sample of emerging adults.

## 5. Conclusions

Despite the limitations, the study is novel in examining the mediating effect of parent-child conflict in the relationship between overparenting and adolescent anxiety using three-wave longitudinal data of Chinese early adolescents in Hong Kong. The findings identified that mother-child conflict mediated the bi-directional associations of maternal overparenting and adolescent anxiety over time, supporting self-determination theory [30]. Moreover, adolescent gender and family intactness did not moderate the relationships among overparenting, parent-child conflict, and adolescent anxiety over time. In view of scant longitudinal research on overparenting in early adolescence, the present study contributes an important step to address the research gaps.

## Figures and Tables

**Table 1:** Descriptive statistics of the variables.

	Range	Mean	SD	Skewness	Kurtosis	Cronbach’s Alpha
1. Perceived paternal overparenting (T1)	1–6	2.59	0.81	0.41	0.49	0.95
2. Perceived paternal overparenting (T2)	1–6	2.56	0.77	0.26	−0.05	0.95
3. Perceived paternal overparenting (T3)	1–6	2.51	0.80	0.34	0.22	0.97
4. Perceived maternal overparenting (T1)	1–6	3.11	0.91	0.07	−0.03	0.95
5. Perceived maternal overparenting (T2)	1–6	3.08	0.90	−0.04	−0.21	0.96
6. Perceived maternal overparenting (T3)	1–6	3.00	0.89	0.13	0.06	0.97
7. Father-child conflict (T1)	1–6	2.72	1.35	0.60	−0.28	0.89
8. Father-child conflict (T2)	1–6	2.86	1.34	0.47	−0.44	0.90
9. Father-child conflict (T3)	1–6	2.92	1.28	0.41	−0.32	0.91
10. Mother-child conflict (T1)	1–6	3.02	1.40	0.36	−0.66	0.90
11. Mother-child conflict (T2)	1–6	3.16	1.32	0.24	−0.52	0.89
12. Mother-child conflict (T3)	1–6	3.23	1.32	0.13	−0.67	0.90
13. Anxiety (T1)	1–4	2.20	0.53	0.31	0.12	0.74
14. Anxiety (T2)	1–4	2.18	0.52	0.25	0.18	0.76
15. Anxiety (T3)	1–4	2.23	0.51	0.26	0.14	0.76
16. Gender (boys = 0, girls = 1)	0–1	N.A.	N.A.	N.A.	N.A.	N.A.
17. Family Intactness (non-intact = 0, intact = 1)	0–1	N.A.	N.A.	N.A.	N.A.	N.A.

**Table 2:** Correlations of the variables.

	1	2	3	4	5	6	7	8	9	10	11	12	13	14	15	16
1. Perceived paternal overparenting (T1)	1.00															
2. Perceived paternal overparenting (T2)	0.61 ***	1.00														
3. Perceived paternal overparenting (T3)	0.50 ***	0.56 ***	1.00													
4. Perceived maternal overparenting (T1)	0.51 ***	0.32 ***	0.28 ***	1.00												
5. Perceived maternal overparenting (T2)	0.34 ***	0.54 ***	0.33 ***	0.63 ***	1.00											
6. Perceived maternal overparenting (T3)	0.30 ***	0.35 ***	0.59 ***	0.54 ***	0.65 ***	1.00										
7. Father-child conflict (T1)	0.32 ***	0.20 ***	0.13 ***	0.17 ***	0.12 ***	0.06 *	1.00									
8. Father-child conflict (T2)	0.20 ***	0.33 ***	0.19 ***	0.05	0.19 ***	0.10 **	0.53 ***	1.00								
9. Father-child conflict (T3)	0.19 ***	0.23 ***	0.32 ***	0.06	0.12 ***	0.19 ***	0.46 ***	0.51 ***	1.00							
10. Mother-child conflict (T1)	0.13 ***	0.06	0.05	0.37 ***	0.27 ***	0.20 ***	0.34 ***	0.16 ***	0.14 ***	1.00						
11. Mother-child conflict (T2)	0.11 ***	0.18 ***	0.10 **	0.27 ***	0.40 ***	0.29 ***	0.23 ***	0.32 ***	0.18 ***	0.51 ***	1.00					
12. Mother-child conflict (T3)	0.07 *	0.07 *	0.19 ***	0.22 ***	0.30 ***	0.40 ***	0.15 ***	0.15 ***	0.35 ***	0.45 ***	0.50 ***	1.00				
13. Anxiety (T1)	0.09 **	0.06	0.04	0.16 ***	0.13 ***	0.10 **	0.22 ***	0.17 ***	0.15 ***	0.29 ***	0.21 ***	0.15 ***	1.00			
14. Anxiety (T2)	0.03	0.06	0.04	0.14 ***	0.19 ***	0.13 ***	0.17 ***	0.21 ***	0.21 ***	0.21 ***	0.26 ***	0.20 ***	0.51 ***	1.00		
15. Anxiety (T3)	0.00	0.04	0.07 *	0.09 **	0.14 ***	0.17 ***	0.12 ***	0.14 ***	0.23 ***	0.17 ***	0.20 ***	0.26 ***	0.41 ***	0.54 ***	1.00	
16. Gender (boys = 0, girls = 1)	−0.09 **	−0.11 **	−0.15 ***	−0.03	−0.07 *	−0.10 **	−0.06	−0.09 **	−0.07 *	−0.01	−0.03	−0.03	−0.02	−0.06 *	0.01	1.00
17. Family Intactness (non-intact = 0, intact = 1)	0.08 **	0.08 **	0.09 **	−0.03	−0.02	−0.01	−0.01	−0.01	0.06	−0.10 **	−0.05	−0.08 **	−0.03	−0.02	−0.06 *	−0.04

* *p* < 0.05, ** *p* < 0.01, *** *p* < 0.001.

**Table 3:** Cross-lagged panel analyses of mediating role of parent-child conflict between overparenting and adolescent anxiety and moderating roles of adolescent gender and family intactness.

Description	Model	*x^2^*	*df*	CFI	NFI	RMSEA	Model Comparison	Δ*x^2^*	Δ*df*
Mediational model of overparenting on adolescent anxiety via parent-child conflict	M1	441.894 ***	66	0.945	0.936	0.073			
Invariant test of M1 by adolescent gender									
Unconstrained model	M2a	471.671 ***	110	0.947	0.934	0.055			
Constrained model	M2b	511.561 ***	145	0.947	0.928	0.049	M2b and M2a	41.564	35
Invariant test of M1 by family intactness									
Unconstrained model	M3a	460.588 ***	110	0.947	0.934	0.056			
Constrained model	M3b	497.189 ***	145	0.947	0.928	0.049	M3b and M3a	40.324	35

*** *p* < 0.001.

## Data Availability

The data presented in this study are available on request from the corresponding author. The data are not publicly available due to privacy.

## References

[1] Burke T.J., Segrin C., Farris K.L. (2018). Young adult and parent perceptions of facilitation: Associations with overparenting, family functioning, and student adjustment. J. Fam. Commun..

[2] Rousseau S., Scharf M. (2015). “I will guide you” The indirect link between overparenting and young adults’ adjustment. Psychiat. Res..

[3] Schiffrin H.H., Liss M., Miles-McLean H., Geary K.A., Erchull M.J., Tashner T. (2014). Helping or hovering? The effects of helicopter parenting on college students’ well-being. J. Child Fam. Stud..

[4] Segrin C., Woszidlo A., Givertz M., Bauer A., Taylor Murphy M. (2012). The association between overparenting, parent-child communication, and entitlement and adaptive traits in adult children. Fam. Relat..

[5] Jiao J., Segrin C. (2021). Parent–emerging-adult-child attachment and overparenting. Fam. Relat..

[6] Segrin C., Woszidlo A., Givertz M., Montgomery N. (2013). Parent and child traits associated with overparenting. J. Soc. Clin. Psychol..

[7] Leung J.T.Y. (2020). Too much of a good thing: Perceived overparenting and wellbeing of Chinese adolescents in Hong Kong. Child Indic. Res..

[8] LeMoyne T., Buchanan T. (2011). Does “hovering” matter? Helicopter parenting and its effect on well-being. Sociol. Spectr..

[9] Perez C.M., Nicholson B.C., Dahlen E.R., Leuty M.E. (2020). Overparenting and emerging adults’ mental health: The mediating role of emotional distress tolerance. J. Child Fam. Stud..

[10] Hong J.C., Hwang M.Y., Kuo Y.C., Hsu W.Y. (2015). Parental monitoring and helicopter parenting relevant to vocational student’s procrastination and self-regulated learning. Learn. Individ. Differ..

[11] Leung J.T.Y., Shek D.T.L., Fung A.L.C., Leung G.S.M. (2020). Perceived overparenting and developmental outcomes among Chinese adolescents: Do family structure and conflicts matter?. J. Soc. Pers. Relat..

[12] Schiffrin H.H., Liss M. (2017). The effects of helicopter parenting on academic motivation. J. Child Fam. Stud..

[13] Odenweller K.G., Booth-Butterfield M., Weber K. (2014). Investigating helicopter parenting, family environments, and relational outcomes for millennials. Commun. Stud..

[14] Reed K., Duncan J.M., Lucier-Greer M., Fixelle C., Ferraro A.J. (2016). Helicopter parenting and emerging adult self-efficacy: Implications for mental and physical health. J. Child Fam. Stud..

[15] Rapee R.M. (2009). Early adolescents’ perceptions of their mother’s anxious parenting as a predictor of anxiety symptoms 12 months later. J. Abnorm. Child Psychol..

[16] Longmore M.A., Manning W.D., Giodano P.C., Fine M.A., Fincham F.D. (2013). Parent-child relationships in adolescence. Handbook of Family Theories: A Content-Based Approach.

[17] Grotevant H.D., Cooper C.R. (1986). Individuation in family relationships—A perspective on individual-differences in the development of identity and role-taking skill in adolescence. Hum. Dev..

[18] Bornstein M.H. (2012). Cultural approaches to parenting. Parent. Sci. Pract..

[19] Yeh M.H., Yang K.S. (1997). Chinese familism: Conceptual analysis and empirical assessment. Bull. Inst. Eth. Acad. Sin..

[20] Leung J.T.Y. (2017). Cultural family beliefs, maternal sacrifice and adolescent psychological competence in Chinese poor single-mother families. Soc. Dev..

[21] Ting K.F., Chiu S.W. (2002). Leaving the parental home: Chinese culture in an urban context. J. Marriage Fam..

[22] Leung J.T.Y., Shek D.T.L., Ng L.S.L. (2018). Over-parenting from the perspectives of Chinese parents and youths. Int. J. Child. Adolesc. Health.

[23] Winner N.A., Nicholson B.C. (2018). Overparenting and narcissism in young adults: The mediating role of psychological control. J. Child Fam. Stud..

[24] Shek D.T.L. (2008). Perceived parental control and parent-child relational qualities in early adolescents in Hong Kong: Parent gender, child gender and grade differences. Sex Roles.

[25] Leung J.T.Y., Shek D.T.L. (2016). The influence of parental beliefs on the development of Chinese adolescents experiencing economic disadvantage: Maternal control as a mediator. J. Fam. Issues.

[26] Qurban H., Wang J., Siddique H., Morris T., Qiao Z. (2019). The mediating role of parental support: The relation between sports participation, self-esteem, and motivation for sports among Chinese students. Curr. Psychol..

[27] Baumrind D., Brooks-Gunn J., Lerner R.A., Peterson C. (1991). Parenting styles and adolescent development. The Encyclopedia of Adolescence.

[28] Leung J.T.Y., Shek D.T.L. (2019). Hierarchical factor analysis and factorial invariance of the Chinese overparenting scale. Front. Psychol..

[29] Bowlby J. (1977). The making and breaking of affectional bonds: I. Aetiology and psychopathology in the light of attachment theory. Brit. J. Psychiat..

[30] Oshino S., Suzuki A., Ishii G., Otani K. (2007). Influences of parental rearing on the personality traits of healthy Japanese. Compr. Psychiat..

[31] Ryan R.M., Deci E.L. (2000). Self-determination theory and the facilitation of intrinsic motivation, social development, and well-being. Am. Psychol..

[32] Erikson E.H. (1968). Identity: Youth and Crisis.

[33] Gavazzi S.M., Sabatelli R.M. (1990). Family system dynamics, the individuation process, and psychosocial development. J. Adolesc. Res..

[34] Leung J.T.Y. (2020). Concerted cultivation and adolescent psychopathology over time—Mediation of parent-child conflict. Int. J. Environ. Res. Pub. Health.

[35] Sameroff A., Sameroff A. (2009). The transactional model. The Transactional Model of Development: How Children and Contexts Shape Each Other.

[36] Leung J.T.Y. (2020). Perceived parental sacrifice, filial piety and hopelessness among Chinese adolescents: A cross-lagged panel study. J. Adolesc..

[37] Reitz E., Deković M., Meijer A.M., Engels R.C. (2006). Longitudinal relations among parenting, best friends, and early adolescent problem behavior: Testing bidirectional effects. J. Early Adolesc..

[38] Willoughby T., Hamza C.A. (2011). A longitudinal examination of the bidirectional associations among perceived parenting behaviors, adolescent disclosure and problem behavior across the high school years. J. Youth Adolesc..

[39] Hudson J.L., Comer J.S., Kendall P.C. (2008). Parental responses to positive and negative emotions in anxious and nonanxious children. J. Clin. Child Adolesc..

[40] Hudson J.L., Rapee R.M. (2001). Parent–child interactions and anxiety disorders: An observational study. Behav. Res. Ther..

[41] Bem S.L. (1974). The measurement of psychological androgyny. J. Consult. Clin. Psychol..

[42] Russell A., Aloa V., Feder T., Glover A., Miller H., Palmer G. (1998). Sex-based differences in parenting styles in a sample with preschool children. Aust. J. Psychol..

[43] Nelson M.K. (2010). Parenting out of Control: Anxious Parenting in Uncertain Times.

[44] Hosley C.A., Montemayor R., Lamb M.E. (1997). Fathers and adolescents. The Role of the Father in Child Development.

[45] Plunkett S.W., Henry C.S., Robinson L.C., Behnke A., Falcon P.C. (2007). Adolescent perceptions of parental behaviors, adolescent self-esteem, and adolescent depressed mood. J. Child Fam. Stud..

[46] Kouros C.D., Pruitt M.M., Ekas N.V., Kiriaki R., Sunderland M. (2017). Helicopter parenting, autonomy support, and college students’ mental health and well-being: The moderating role of sex and ethnicity. J. Child Fam. Stud..

[47] Janssens K.A.M., Oldehinkel A.J., Rosmalen J.G.M. (2009). Parental overprotection predicts the development of functional somatic symptoms in young adolescents. J. Pediatr..

[48] Darlow V., Norvilitis J.M., Schuetze P. (2017). The relationship between helicopter parenting and adjustment to college. J. Child Fam. Stud..

[49] Scharf M., Rousseau S., Bsoul S. (2017). Overparenting and young adults’ interpersonal sensitivity: Cultural and parental gender-related diversity. J. Child Fam. Stud..

[50] Erel O., Burman B. (1995). Interrelatedness of marital relations and parent–child relations: A meta-analytic review. Psychol. Bull..

[51] Cummings E.M., Merrilees C.E., George M.W., Lamb M. (2010). Fathers, marriages and families: Revisiting and updating the framework for fathering in family context. The Role of the Father in Child Development.

[52] Yoo J. (2020). Relationships between Korean parents’ marital satisfaction, parental satisfaction, and parent–child relationship quality. J. Soc. Pers. Relat..

[53] Brenning K.M., Soenens B., Van Petegem S., Kins E. (2017). Searching for the roots of overprotective parenting in emerging adulthood: Investigating the link with parental attachment representations using an actor partner interdependence model (APIM). J. Child Fam. Stud..

[54] Villalobos A. (2015). compensatory connection: Mothers’ own stakes in an intensive mother-child relationship. J. Fam. Issues.

[55] Census and Statistics Department (2021). Hong Kong Monthly Digest of Statistics: Statistics on Comprehensive Social Security Assistance Scheme, 2010 to 2020 (Feature Article).

[56] Census and Statistics Department (2021). Hong Kong Annual Digest of Statistics.

[57] Fitzmaurice G.M., Heath A.F., Clifford P. (1996). Logistic regression models for binary panel data with attrition. J. R. Stat. Soc. Ser. A Stat. Soc..

[58] Leung J.T.Y., Shek D.T.L. (2018). Validation of the perceived Chinese overparenting scale in emerging adults in Hong Kong. J. Child Fam. Stud..

[59] Shek D.T.L. (1998). A longitudinal study of the relations between parent-adolescent conflict and adolescent psychological well-being. J. Genet. Psychol..

[60] Robin A.L., Foster S.L. (1989). Negotiating Parent-Adolescent Conflict.

[61] Shek D.T.L. (2002). The relation of parental qualities to psychological well-being, school adjustment and problem behavior in Chinese adolescents with economic disadvantage. Am. J. Fam. Ther..

[62] Zigmond A.S., Snaith R.P. (1983). The hospital anxiety and depression scale. Acta Psychiat. Scand..

[63] Leung C.M., Ho S., Kan C.S., Hung C.H., Chen C.N. (1993). Evaluation of the Chinese version of the hospital anxiety and depression scale. A cross-cultural perspective. Int. J. Psychosom..

[64] Arbuckle J.L. (2007). AMOS 16.0 User’s Guide.

[65] Hu L.T., Bentler P.M. (1999). Cutoff criteria for fit indexes in covariance structure analysis: Conventional criteria versus new alternatives. Struct. Equ. Modeling.

[66] Preacher K.J., Hayes A.F. (2008). Asymptotic and resampling strategies for assessing and comparing indirect effects in multiple mediator models. Behav. Res. Methods.

[67] MacKinnon D.P. (2008). Mediation analysis. Annu. Rev. Psychol..

[68] Alwin D.F., Hauser R.M. (1975). Decomposition of effects in path analysis. Am. Sociol. Rev..

[69] Curran P.J., West S.G., Finch J.F. (1996). The robustness of test statistics to nonnormality and specification error in confirmatory factor analysis. Psychol. Methods.

[70] Enders C.K. (2010). Applied Missing Data Analysis (Methodology in the Social Sciences).

[71] Enders C.K. (2001). The performance of the full information maximum likelihood estimator in multiple regression models with missing data. Educ. Psychol. Meas..

[72] Givertz M., Segrin C. (2014). The association between overinvolved parenting and young adults’ self-efficacy, psychological entitlement, and family communication. Commun. Res..

[73] Kwon S.H., Lee J. (2014). The relationship between maternal and paternal parenting styles and young adults’ career decision-making: The mediational roles of differentiation of self. J. Korean Home Manag. Assoc..

[74] Eagly A.H. (1987). Sex Differences in Social Behavior: A Social-Role Interpretation.

[75] Leung J.T.Y., Shek D.T.L. (2011). “All I can do for my child”—development of the Chinese parental sacrifice for child’s education scale. Int. J. Disabil. Hum. Dev..

[76] Hudson J.L., Rapee R.M., Heimberg R.G., Turk C.L., Mennin D.S. (2004). From anxious temperament to disorder: An etiological model of Generalized Anxiety Disorder. Generalized Anxiety Disorder: Advances in Research and Practice.

[77] Steinberg L. (2001). We know some things: Parent–adolescent relationships in retrospect and prospect. J. Res. Adolesc..

[78] Erozkan A. (2012). Examination of relationship between anxiety sensitivity and parenting styles in adolescents. Educ. Sci. Theor. Pract..

[79] Ginsburg G., Siqueland L., Masia-Warner C., Hedtke K. (2004). Anxiety disorders in children: Family matters. Cogn. Behav. Pract..

[80] Hale III W.W., Engels R., Meeus W. (2006). Adolescent’s perceptions of parenting behaviours and its relationship to adolescent generalized anxiety disorder symptoms. J. Adolesc..

[81] Schleider J.L., Vélez C.E., Krause E.D., Gillham J. (2014). Perceived psychological control and anxiety in early adolescents: The mediating role of attributional style. Cogn. Ther. Res..

[82] Elstad J.I., Stefansen K. (2014). Social variations in perceived parenting styles among Norwegian adolescents. Child Indic. Res..

[83] Day R.D., Gavazzi S., Acock A., Thornton A. (2001). Compelling family processes. The Well-Being of Children and Families—Research and Data Needs.

[84] Liu J., Zhou T., Yuan M., Ren H., Bian X., Coplan R.J. (2021). Daily routines, parent–child conflict, and psychological maladjustment among Chinese children and adolescents during the COVID-19 pandemic. J. Fam. Psychol..

